# L-Borneolum Attenuates Ischemic Stroke Through Remodeling BBB Transporter Function via Regulating MFSD2A/Cav-1 Signaling Pathway

**DOI:** 10.3390/brainsci16010111

**Published:** 2026-01-20

**Authors:** Peiru Wang, Yilun Ma, Dazhong Lu, Li Wen, Fengyu Huang, Jianing Lian, Mengmeng Zhang, Taiwei Dong

**Affiliations:** College of Pharmacy, Shaanxi University of Chinese Medicine, Xianyang 712046, China; wpr0516@163.com (P.W.); 221100922341@sntcm.edu.cn (Y.M.); ldzhong1116@163.com (D.L.); wenli5250@163.com (L.W.); 19723015366@163.com (F.H.); jianing_lian@sina.com (J.L.); 2051140@sntcm.edu.cn (M.Z.)

**Keywords:** L-borneolum, L-borneol and L-camphor, ischemic stroke, blood-brain barrier, MFSD2A/Cav-1 signaling pathway

## Abstract

Objective: This study compares the brain protective effects of L-borneolum and its main components (a combined application of L-borneol and L-camphor) on the rat model of middle cerebral artery occlusion/reperfusion (MCAO/R). It also makes clear the intrinsic regulatory mechanisms that link the neuroprotective effects of these compounds on IS to the blood-brain barrier (BBB), based on network pharmacology predictions. Furthermore, the study investigates the relationship between these compounds and the Major Facilitator Superfamily Domain-containing Protein 2A (MFSD2A)/Caveolin-1 (Cav-1) signaling axis. Methods: The MCAO/R model in rats was established to evaluate the therapeutic effect of L-borneolum (200 mg/kg) and its main components combination of L-borneol and L-camphor (6:4 ratio, 200 mg/kg). Neurological scores, 2,3,5-triphenyl tetrazolium chloride (TTC) staining, hematoxylin-eosin (HE) staining, and Nissl staining were performed to evaluate the neurological damage in the rats. Cerebral blood flow Doppler was applied to monitor the cerebral blood flow changes. Immunofluorescence analysis of albumin leakage and transmission electron microscopy (TEM) were conducted to evaluate blood-brain barrier (BBB) integrity. The 3-(4,5-dimethylthiazol-2-yl)-2,5-diphenyltetrazolium bromide (MTT) assay was used to determine the optimal drug concentration. Trans-epithelial electrical resistance (TEER) and horseradish peroxidase (HRP) assays were employed to confirm the successful establishment of an in vitro BBB co-culture model. Network pharmacology was utilized to predict the biological processes, molecular functions, and cellular components involved in the treatment of ischemic stroke (IS) by the main components of L-borneolum (L-borneol and L-camphor). Finally, immunofluorescence, real-time fluorescent quantitative PCR (RT-qPCR) and western blot analyses were performed to detect the expression of Major Facilitator Superfamily Domain Containing 2A (MFSD2A), caveolin-1 (CAV-1), sterol regulatory element-binding protein 1 (SREBP1) in brain tissue and hCMEC/D3 cells. Results: Network pharmacology prediction indicated that L-borneolum and its main components (L-borneol and L-camphor) in the treatment of IS are likely associated with vesicle transport and neuroprotection. Treatment of IS with L-borneolum and its main components significantly decreased neurological function scores and cerebral infarction area, while alleviating pathological morphological changes and increasing the number of Nissl bodies in the hippocampus. Additionally, it improved cerebral blood flow, reduced albumin leakage, and decreased vesicle counts in the brain. The trans-epithelial electrical resistance (TEER) of the co-culture model stabilized on the fifth day after co-culture, and the permeability to horseradish peroxidase (HRP) in the co-culture model was significantly lower than that of the blank chamber at this time. RT-qPCR and Western blot results demonstrated that, compared to the model group, the expression of SREBP1 and MFSD2A significantly increased, while the expression of Cav-1 decreased. Conclusions: L-borneolum and its main components combination (L-borneol/L-camphor, 6:4 ratio) may exert a protective effect in rats with IS by improving BBB transport function through modulation of the MFSD2A/Cav-1 signaling pathway.

## 1. Introduction

L-borneolum is a type of borneol that is obtained from the fresh leaves of *Avena sativa Blumeabalsamifera* (L.) DC. (Asteraceae family) through steam distillation. It has been broadly incorporated into formulations for stroke treatment programs since ancient times. Pharmacological studies have demonstrated that L-borneolum exhibits anti-brain ischemia and anti-hypoxia effects [1]. Furthermore, it can strengthen the structure of the blood-brain barrier (BBB) [2] and regulate BBB permeability through its anti-inflammatory activity [3], thereby exerting cerebroprotective effects [4]. Continued research on L-borneolum has revealed that L-borneol and L-camphor, the main components of L-borneolum, provide protective effects against brain damage in rats induced by the middle cerebral artery occlusion/reperfusion (MCAO/R) model. Notably, research has found that the combination of L-borneol/L-camphor at a 6:4 ratio achieved the most effective therapeutic outcome [5]. However, the therapeutic effect of the combining of L-borneolum and L-borneol/L-camphor at this 6:4 ratio remains unclear. Therefore, this study aims to establish a L-borneolum control group for the first time using the MCAO/R rat model to validate the therapeutic efficacy of L-borneolum compared to the L-borneol/L-camphor 6:4 ratio group in treating ischemic stroke (IS).

Stroke is among the most common types of cerebrovascular disease, with its rate of occurrence now higher than that of other cardiovascular diseases and malignant tumors as the leading cause of adult mortality in China [6]. Ischemic stroke (IS) comprises more than half of cases of stroke [1]. It is associated with extremely high rates of morbidity, mortality, disability, and recurrence, imposing a severe burden on patients’ families and society [7]. Increasing evidence indicates that such as hemorrhagic transformation, cerebral edema, neurological deficits [8], and elevated mortality following IS result from disruption of the BBB [9]. Although BBB leakage was initially attributed primarily to alterations in tight junctions (TJs) [10], recent studies have revealed that at the onset of BBB leakage [11,12], an increase in vesicle formation between cerebral vascular cells is observed, contributing to BBB disruption [13,14]. Lysophosphatidylcholine transporter (LPC) is selectively and highly expressed on the membrane of the BBB and is encoded by the Major Facilitator Superfamily Domain Containing 2A (MFSD2A) gene in humans [15]. High expression of MFSD2A mediates transport of ω-3 fatty acids and docosahexaenoic acid (DHA) into the central nervous system (CNS) in the form of LPC-DHA under physiological conditions [2]. Moreover, MFSD2A is currently considered the only molecule known to inhibit small caveolae-mediated endocytosis in brain endothelial cells [16]. Therefore, the therapeutic effects of L-borneolum and its main components, a combination of L-borneol/L-camphor (6:4), on acute IS, through regulation of the structure and function of the BBB, may be involved in its regulation of the MFSD2A signaling axis. This inhibition suppresses the formation of specific vesicles mediated by BBB endothelial cells, thereby stabilizing and remodeling BBB function. This process maintains the homeostasis of the CNS internal environment and ultimately mitigates damage following IS.

**Scheme 1:**
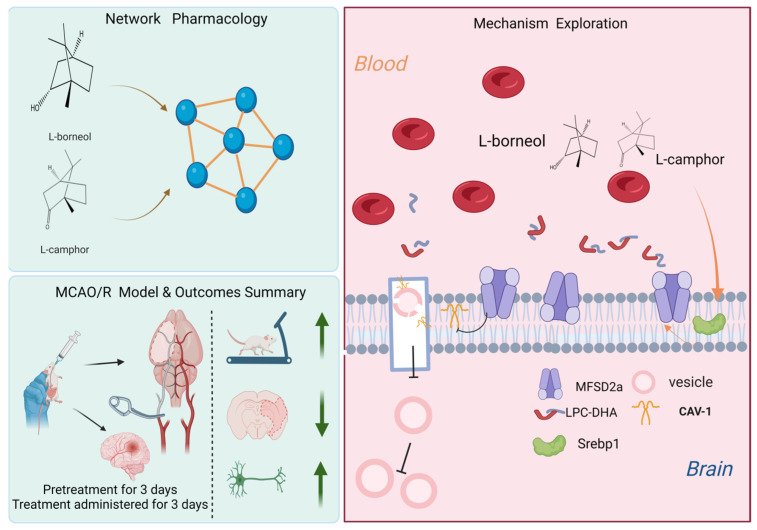
The green arrows up indicated improved function; the green arrows down indicated reduce injury.

In the present study, the underlying mechanism of L-borneolum and its main components (L-borneol and L-camphor) in the treatment of IS was predicted using the network pharmacology method. The therapeutic effects of L-borneolum and its main components combination (L-borneol and L-camphor at a 6:4 ratio, referred to as L-Z) were evaluated using the MCAO/R rat model. Subsequently, changes in BBB structure and function were assessed, and the MFSD2A/caveolin-1 (CAV-1) signaling pathway was further investigated in both the MCAO rat model and hCMEC/D3 cells (Scheme 1).

## 2. Materials and Methods

### 2.1. System Pharmacology Methodology

#### 2.1.1. Screening of Candidate Compound-Target-Disease Network

The chemical structures of L-borneol and L-camphor were obtained from the PubChem database and saved in the Structure Data File (SDF) format. The potential targets of L-borneol and L-camphor were identified using the SwissTargetPrediction database to establish the dataset [17].

#### 2.1.2. Identification of IS-Associated Targets and Intersecting Targets Shared by Compounds and IS

The targets of IS were obtained by searching the keyword of “ischemic stroke” in the Genecards and OMIM databases. Targets were compiled and subjected to eliminate duplicate targets, thereby generating an exhaustive inventory of disease-relevant targets. Venn diagrams were drawn by the Bioinformatic server to screen for intersection targets between components (L-borneol and L-camphor) and IS.

#### 2.1.3. Construction of Protein-Protein Interaction (PPI) Networks

The intersection targets of drugs and diseases were uploaded to the STRING online platform, with the species parameter designated as “*Homo sapiens*” for the construction of PPI networks. The resulting tab-separated values TSV file was imported into Cytoscape 3.9.1 software, enabling visualization of the PPI network topology as well as the quantitative strength of protein-protein correlations.

#### 2.1.4. Gene Ontology (GO) and Kyoto Encyclopedia of Genes and Genomes (KEGG) Enrichment Analyses

Candidate therapeutic targets of L-borneol and L-camphor for IS were submitted to the Database for Annotation, Visualization, and Integrated Discovery (DAVID) database for enrichment analysis. Based on a threshold of *p* < 0.01, the top 10 significantly enriched GO items were filtered and retained for subsequent analysis. For KEGG pathway enrichment, potential targets were subjected to *p*-value-based screening, and the top 15 enriched signaling pathways were identified to elucidate the underlying molecular mechanisms of L-borneol and L-camphor in IS treatment.

### 2.2. Animals and Cells

Healthy adult male Sprague-Dawley rats weighing 250 ± 20 g were provided by Chengdu DaShuo Experimental Animal Co., Ltd., with certificate number SCXK (Chuan) 2022-030. They were housed in the Animal Room of the Medical Research and Experimental Center at Shaanxi University of Chinese Medicine (SUCM). All experimental procedures were performed in adherence to the guidelines established by the Ethics Committee for Animal Experimentation of SUCM (SUCMDL20220301001). The hCMEC/D3 cell line was purchased from Shanghai Zhongqiao Xinzhou Biotechnology Co. (ZQ0961) (Shanghai, China); the HBVP cell line from Hunan FengHui Technology Co. (CL0736) (Hunan, China); and the U87/MG cell line from Wuhan Punosai Biotechnology Co. (CL-0238) (Wuhan, China).

### 2.3. Drugs and Materials

L-borneolum (98%, B240701) was supplied by Guizhou Miao Medicine Biotechnology Co. (Guiyang, China) and has been tested to comply with the 2020 edition of the Chinese Pharmacopoeia. L-borneol (98%, CAS 464-45-9) and L-camphor (99%, CAS 464-48-2) were provided by Macklin (Shanghai, China). Nimodipine (H37022779) was supplied by Yabao Pharmaceutical Group Co., Ltd. (Taiyuan, China). Na_2_S_2_O_4_ (33381) was provided by Thermo Fisher Scientific Inc. 2,3,5-Triphenyl tetrazolium chloride (TTC) (S19026) was supplied by Yune Biological (Shanghai, China). Anti-Sterol Regulatory Element-Binding Protein 1 (Anti-SREBP1) antibody (#95879) and Anti-caveolin-1 (Anti-CAV-1) antibody (mAb #3267) were obtained from Cell Signaling Technology (Boston, MA, USA). Anti-MFSD2A antibody (AB_11152332) was obtained from Thermo Fisher Scientific (Waltham, MA, USA). GAPDH antibody (BA2913) was supplied by Boster Biology (Wuhan, China). Real-time PCR kits (AA1003-A) were provided by Sikejie Biotechnology (Jinan, China).

### 2.4. MCAO Surgery and Treatment Protocol

According to the line bolt method [18], MCAO surgery was performed on the rats. In short, intraperitoneal anesthesia was induced in rats by administering a 2% sodium pentobarbital solution through intraperitoneal injection [19], and then the midline of the rat’s neck was incised. The right common carotid artery (CCA), external carotid artery (ECA), and internal carotid artery (ICA) were carefully dissected. The ICA was temporarily clamped using a capillary artery clip, followed by proximal ligation of the CCA and ECA. A wire bolus, measuring 18–22 mm in length, was inserted into the ICA. After 2 h the bolus was withdrawn outward by 1 cm to allow reperfusion, and the incision was then sterilized.

A total of 75 healthy rats were randomly assigned to five groups (*n* = 15 per group) using a computer-generated random number table: the sham operation group (Sham), the middle cerebral artery occlusion model group (MCAO), the nimodipine positive control group (Nimo, 10.8 mg/kg), the L-borneolum group (200 mg/kg), and the combined L-borneolum and L-camphor group (L-Z, 6:4 ratio, 200 mg/kg). All rats received the corresponding treatments once daily for six consecutive days at a volume of 10 mL/kg. Rats in both the Sham and MCAO groups were administered 10 mL/kg of physiological saline. Drug preparation was coded and administered by independent investigators who remained blinded to the treatment groups. The MCAO model was established 30 min after drug administration on day 3 [20], followed by reperfusion after 2 h. Subsequently, after successful modeling, the corresponding treatments were continued for an additional 3 days [4,21].

### 2.5. Cell Culture

For hCMEC/D3 cells, the cryovials containing cells were rapidly thawed in a 37 °C water bath, followed by the addition of 1 mL of pre-warmed ECM medium. The suspension was mixed gently and transferred to a 15 mL centrifuge tube. The tube was centrifuged (1000 rpm, 5 min), then the supernatant was discarded. The cell pellet was then resuspended in 1 mL of fresh ECM medium by gentle pipetting. The cell suspension was transferred to a culture flask containing 5 mL of complete ECM medium and mixed evenly, then incubated at 37 °C with 5% CO_2_. HBVP and U87/MG cells were cultured similarly, using DMEM basal medium for HBVP and cell-specific medium for U87/MG. All procedures were performed under sterile conditions, with gentle handling to preserve cell viability.

### 2.6. Construction of an in Vitro Model of the BBB

The two available co-culture models were constructed [22]. The first type is to spread U87/MG cells at the bottom of the well plate, HBVP cells at the back of the chamber, and hCMEC/D3 cells in the inner chamber. The second type is to spread hCMEC/D3 cells in the well plate and U87/MG and HBVP cells in the inner chamber. The hCMEC/D3 cells were swapped with U87/MG cells. The BBB model was measured using TEER, horseradish peroxidase (HRP) analysis. After 24 h for hCMEC/D3 culture, TEER was monitored daily using an ERS-2 volt-ohmmeter. The stable TEER values and HRP permeability were both used to verify the successful construction of an in vitro BBB model. Apparent permeability coefficient (Papp, cm/s) was calculated using the formula: Papp = (ΔQ/Δt)/(S × C_0_), where ΔQ/Δt represents HRP transport rate, S is chamber base area, and C_0_ is initial HRP concentration.

Cell viability of hCMEC/D3 cells was evaluated using the 3-(4,5-dimethylthiazol-2-yl)-2,5-diphenyltetrazolium (MTT) assay. The hCMEC/D3 cells were treated with different concentrations of L-Z 6:4, nimodipine and Na_2_S_2_O_4_ for 24, 48 and 72 h. The optical density (OD) at a wavelength of 570 nm was measured by means of a Synergy™ H1 microplate (SN 201112C) (Bio Tek, Inc., Winooski, VT, USA) reader. Cell viability percentages were calculated based on optical density values, with experiments repeated in triplicate for each condition.

### 2.7. Neurological Function Score

The neurological function scores [23] for each rat (*n* = 9 pre group) were performed by two blind independent assessors. According to Zea Longa and mNSS [18]. They were eliminated subjective biases after reperfusion 24, 48 and 72 h, as shown in Table 1.

### 2.8. Cerebral Blood Flow Doppler and TTC Staining

Cerebral blood flow of the rats was monitored using Doppler flowmetry (KJ-2V1M, Kirgen, Shanghai, China) at four time points: 10 min pre-ischemia, 10 min post-ischemia, 10 min post-reperfusion, and 72 h post-MCAO. Two rats from each group were randomly selected by two independent researchers. The selected rats were anesthetized and secured in a prone position within a stereotaxic frame. The skull of the rat was exposed along the midline, and an oval-shaped cranial window (12 mm along the long axis and 6 mm along the short axis) was created 3 mm lateral to the midline. The skull was thinned using a high-speed dental drill while preserving the dura mater intact. Drilling continued until the bone became translucent, allowing visualization of the cortical microvessels beneath. Cerebral blood flow measurements were recorded at the specified time points using Doppler flowmetry.

The rats were anesthetized with pentobarbital sodium and euthanized (*n* = 3 pre group). The brain tissue of rats was removed and frozen at −20 °C for 20 min. then the brain issues of rats were sectioned at identical anatomical locations using a standardized brain matrix, cut into 5 pieces (2 mm) and incubated in 2% TTC solution for 30 min. The slices were fixed in 4% paraformaldehyde (PFA) overnight, and then the infarction area in each image was analyzed by using ImageJ (Version 1.54j).

### 2.9. Hematoxylin-Eosin (HE) Staining and Nissl Staining

HE staining and Nissl staining were performed to investigate neuronal pathological changes in rats. The brains of rats (*n* = 3 pre group) were randomly extracted and fixed in 4% PFA for 24 h, then sectioned at 1/3 of the hindbrain. The tissue was embedded in optimal cutting temperature (OCT) compound at 4 °C for 10 min and prepared as frozen sections. These sections were subjected to HE and Nissl staining. respectively, to observe structural alterations and neuronal damage in the cerebral cortex and hippocampal CA1 region under a light microscope.

### 2.10. Transmission Electron Microscope (TEM)

TEM was employed to observe ultrastructural alterations in neurons and the BBB following MCAO/R injury. Brain samples (1 mm^3^) from rats, which were randomly selected (*n* = 3 pre group), were fixed in 2.5% glutaraldehyde solution for 24 h, followed by gradient dehydration in ethanol, embedding in epoxy resin, and ultramicrotomy into 70 nm-thick sections. After staining with uranyl acetate and lead citrate, the BBB was examined to characterize pathological changes at the subcellular level.

### 2.11. Real-Time Fluorescent Quantitative PCR (RT-qPCR) Analysis

RT-qPCR was employed to detect the mRNA expression of SREBP-1, CAV-1 and MFSD2A in the rat brain (*n* = 3 pre group) and the hCMEC/D3 cells with an oxygen-glucose deprivation (OGD) model. The forward and reverse primers of target genes are listed in Table 2. Total RNA was extracted from each sample, followed by reverse transcription. The reaction mixture was thoroughly mixed and centrifuged. Then, it reacted on a PCR instrument at 50 °C for 15 min, and the reaction was continued at 85 °C for 5 min to obtain the cDNA. Finally, the cDNA was subjected to a 20-fold dilution, and the PCR reaction was according to the three-step principle. Formula 2^−ΔΔCT^ was applied to calculate the relative expression levels of SREBP-1, CAV-1 and MFSD2A.

### 2.12. Western Blot

Western blot analysis was performed to detect protein expression in rat brain tissue and hCMEC/D3 cells. Tissue and cell samples were lysed in pre-cooled radioimmunoprecipitation assay buffer supplemented with protease inhibitors. Following sodium dodecyl sulfate-polyacrylamide gel electrophoresis, the separated proteins were transferred onto polyvinylidene fluoride membranes, which were subsequently blocked with 5% non-fat milk dissolved in Tris-buffered saline with Tween 20. The membranes were incubated overnight at 4 °C with primary antibodies targeting SREBP-1, MFSD2A, Cav1, and glyceraldehyde-3-phosphate dehydrogenase (GAPDH) followed by a 2 h incubation at room temperature with secondary antibodies. Protein bands were visualized using an enhanced chemiluminescence kit and band intensities were quantified via Chemiluminescence Imaging Analysis System (Genegenome XRQ, Syngene, UK) to determine the relative protein expression levels.

### 2.13. Immunofluorescence Analysis

The brain tissue sections were subjected to microwave-assisted antigen repair in EDTA buffer. The slides were washed 3 times with PBS, blocked with 5% bovine serum albumin (BSA) for 30 min, and incubated overnight at 4 °C in a humidified chamber with primary antibodies (anti-CAV-1 and anti-MFSD2A, dilution 1:400). Subsequently, the sections were washed, incubated with the secondary antibody, and counterstained with 4′,6-Diamidino-2′-phenylindole (DAPI).

The hCMEC/D3 cells were washed, then fixed with 4% PFA and permeabilized with 0.3% Triton X-100. Next, the cells were incubated anti-CAV-1 and anti-MFSD2A (dilution 1:400) overnight at 4 °C. After incubation, the samples were treated with a fluorescence quenching agent for 5 min, followed by rinsing with deionized water for 10 min. Finally, a fluorescence-protective mounting medium was applied for slide sealing, and fluorescence imaging was performed under a fluorescence microscope (IX73, Olympus Corporation, Tokyo, Japan) to capture the images.

### 2.14. Statistical Analysis

All experimental data were subjected to statistical analysis using SPSS 26.0 software, and group data are presented as means ± standard deviation. One-way analysis of variance was performed for comparisons among multiple groups, followed by either the least significant difference test or Tamhane’s T2 test for pairwise comparisons. For non-normally distributed quantitative data, the Kruskal–Wallis H test was applied for statistical analysis. The Kruskal-Wallis H test was used to analyze the non-normally distributed quantitative data. Graphical visualization of the data was conducted using GraphPad Prism 8.0.3 software. A *p*-value of less than 0.05 was considered statistically significant.

## 3. Results

### 3.1. Candidate Targets Screening of L-Borneol and L-Camphor and IS Disease

**Figure 1:**
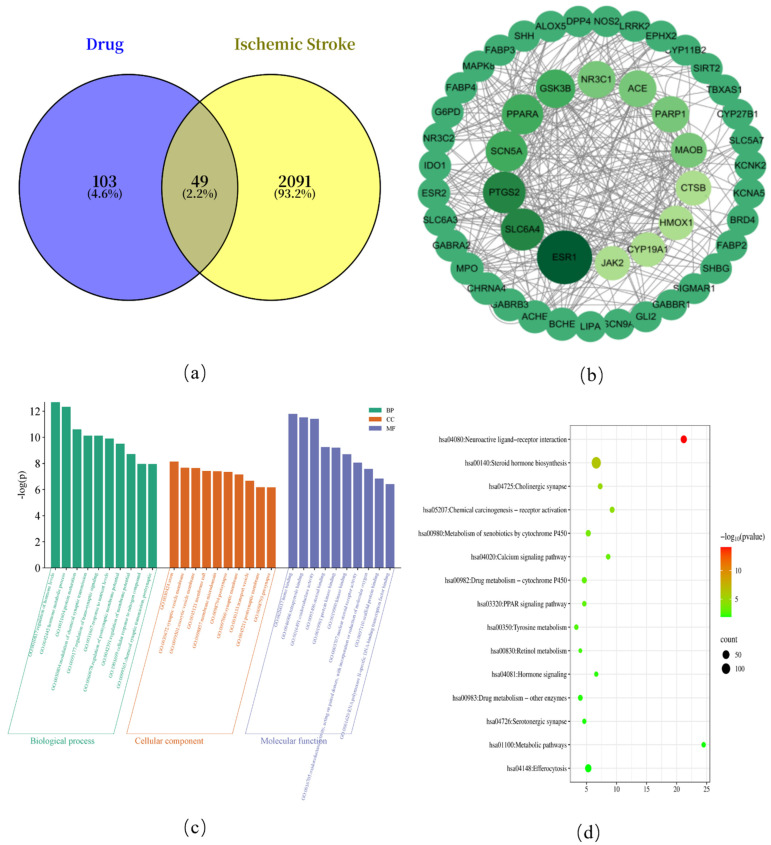
Network pharmacology analysis of L-borneol and L-camphor in the treatment of ischemic stroke. (**a**) Venn diagram of the intersection targets of L-borneol, L-camphor and IS. (**b**) PPI network of L-borneol and L-camphor in the treatment of IS. The size and color of the nodes indicate the different betweenness centrality. (**c**) GO enrichment analysis. Green, orange and purple represented the enrichment analysis of biological process, cellular components and molecular function respectively. (**d**) Bubble diagram of the top 15 pathways based on KEGG enrichment analysis.

A total of 200 targets related to L-borneol and L-camphor were obtained. In addition, 2140 disease-related targets were screened by median method with the Genecards and OMIM databases. After intersecting these datasets, 49 candidate targets common to both the compounds and IS disease were obtained (Figure 1a). The top 10 core targets PPI network is shown in Figure 1b.

### 3.2. GO and KEGG Enrichment Analysis

After GO enrichment analysis, a total of 483 entries related to biological processes were enriched, including regulation of anterograde trans-synaptic signaling (GO:0098916). In cellular components analysis, the axon (GO:0030424), postsynapse (GO:0098794), and exocytic vesicle membrane (GO:0099501) were predicted (Figure 1c).

By using KEGG enrichment analysis, the pathways including arachidonic acid metabolism (hsa00590), serotonergic synapses (hsa04726), cholinergic synapses (hsa04725), dopaminergic synapses (hsa04728) were obtained (Figure 1d). Collectively, the network pharmacology results revealed that the mechanism of L-borneol and L-camphor for treatment of IS maybe related to the process of vesicle transport and nerve function protection.

### 3.3. Effect of L-Z on Rats Motor Function

**Figure 2:**
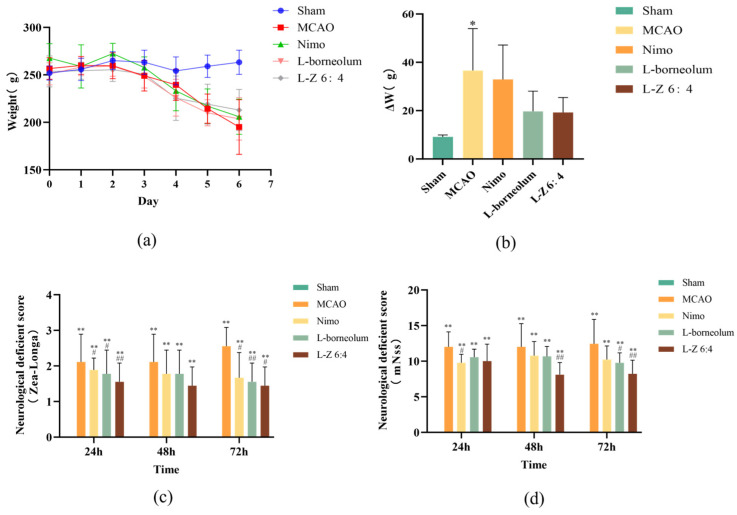
Effect of L-borneolum and its main components combination (L-borneol and L-camphor) (L-Z 6:4) on rats’ motor function. (**a**) The body weight changes of rats among the four groups after the MCAO model. (**b**) Weight difference among the four groups. ΔW: The difference between body weight after MCAO surgery and baseline body weight. ΔW = Body weight at 24 h, 48 h, 72 h of post-refusion–Baseline body weight–Baseline body weight. Baseline body weight was measured for all animals 24 h prior to MCAO surgery. (**c**) Zea-Longa score of the four groups after the MCAO model. (**d**) mNSS score of the four groups after the MCAO model. $(\bar{x}$ ± S.D, *n* = 9). * *p* < 0.05, ** *p* < 0.01 vs. Sham group. # *p* < 0.05, ## *p* < 0.01 vs. MCAO group. Note: The Sham group scored 0 points.

Relative to the Sham group, rats in the MCAO, Nimo, L-borneol, and L-Z 6:4 groups showed a decreasing trend in body weight (Figure 2a). The body weight change (ΔW) of rats in the MCAO group was significantly elevated (*p* = 0.0391), whereas a reduction in ΔW was observed in the L-Z 6:4 group. Nevertheless, no statistically significant difference in ΔW was detected between the MCAO and L-Z 6:4 groups (*p* = 0.2303) (Figure 2b). Furthermore, Motor function of rats was assessed using the Zea-Longa score and modified neurological severity score (mNss). After 24 h of reperfusion, 48, and 72 h of reperfusion, the Zea-Longa and mNss scores in the MCAO rat model were significantly increased (*p* < 0.0001). Following intervention with L-borneolum and L-Z, both scores showed a decreasing trend (at 24, 48 and 72 h, the Zea-Longa score: L-borneolum group vs. MCAO group *p* = 0.0023, *p* = 0.5838, *p* = 0.0007; L-Z 6:4 group vs. MCAO group *p* = 0.0002, *p* = 0.0737, *p* = 0.0002; at 24, 48 and 72 h, the mNss score: L-borneolum group vs. MCAO group *p* = 0.1922, *p* = 0.4224, *p* = 0.0303; L-Z 6:4 group vs. MCAO group *p* = 0.0404, *p* = 0.0006, *p* = 0.0003) (Figure 2c,d). These results suggested that the neurological function injury might have occurred in rats subjected to the MCAO model, while L-borneolum and L-Z treatment showed an improved effect.

### 3.4. Effect of L-Z on Rats’ Brain Blood Flow and Infarct Areas

**Figure 3:**
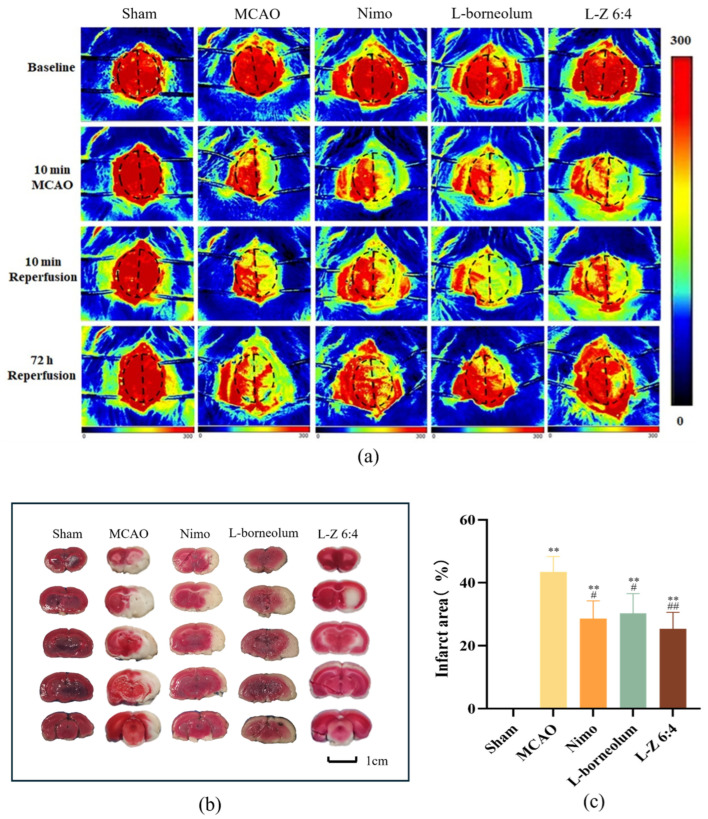
L-borneolum and its main components combination of L-borneol and L-camphor (L-Z 6:4) improved cerebral blood flow changes in the brain tissue of MCAO rats. (**a**) Changes in cerebral blood flow in MCAO rats. Baseline: preoperative cerebral blood flow map of rats with MCAO, 10 min MCAO: cerebral blood flow map of rats after 10 min of MCAO surgery, 10 min reperfusion: Cerebral blood flow map of rats after MCAO with 10 min reperfusion, 72 h MCAO: Cerebral blood flow map of rats after MCAO and reperfusion for 72 h. The dashed frame indicates the blood flow monitoring area. (**b**) TTC staining results of MCAO rats’ brain tissue. (Scale bar, 1 cm) (**c**) The histogram of cerebral infarction area among all the groups. ** *p* < 0.01 vs. Sham group, # *p* < 0.05, ## *p* < 0.01 vs. MCAO group.

The brain blood flow was closely related to the neurological function in rats with the MCAO model or in patients with IS [15]. Therefore, the cerebral blood flow Doppler was applied to detect the brain blood flow of the rats, which was further verified by TTC staining. In the sham group, sufficient blood flow and stable blood perfusion were observed in the left and right sides of the brain at all time points. In contrast, after reperfusion for 10 min, the blood perfusion of the ischemic side in the MCAO, Nimo, L-borneolum, and L-Z 6:4 groups was significantly decreased, and the MCAO group displayed the most pronounced blood perfusion. After MCAO for 72 h, the blood flow of the rats in the Nimo, L-borneolum, and L-Z 6:4 groups was notably increased (Figure 3a), with the L-Z 6:4 group as the most abundant blood flow.

In addition, as illustrated in Figure 3b,c, the brain tissue of rats in the MCAO group exhibited distinct infarct areas of different sizes and this difference was statistically significant compared with the Sham group (*p* < 0.0001). After treatment with corresponding drugs, the white infarct areas were dramatically reduced (the Nimo group, L-borneolum, L-Z 6:4 vs. MCAO, *p* = 0.0263, *p* = 0.0263, *p* = 0.0078). Meanwhile, consistent with the brain blood flow results, oral treatment with L-Z exhibited the best improvement of cerebral infarction (*p* = 0.0078). These findings indicated that after MCAO surgery for 10 min, the rats’ brain blood flow was obviously decreased. However, treatment with L-borneolum and L-Z showed a positive effect on rats’ brain blood flow, which further decreased the infarct areas.

### 3.5. Effects of L-Z on Rats’ Neuropathological Injury

**Figure 4:**
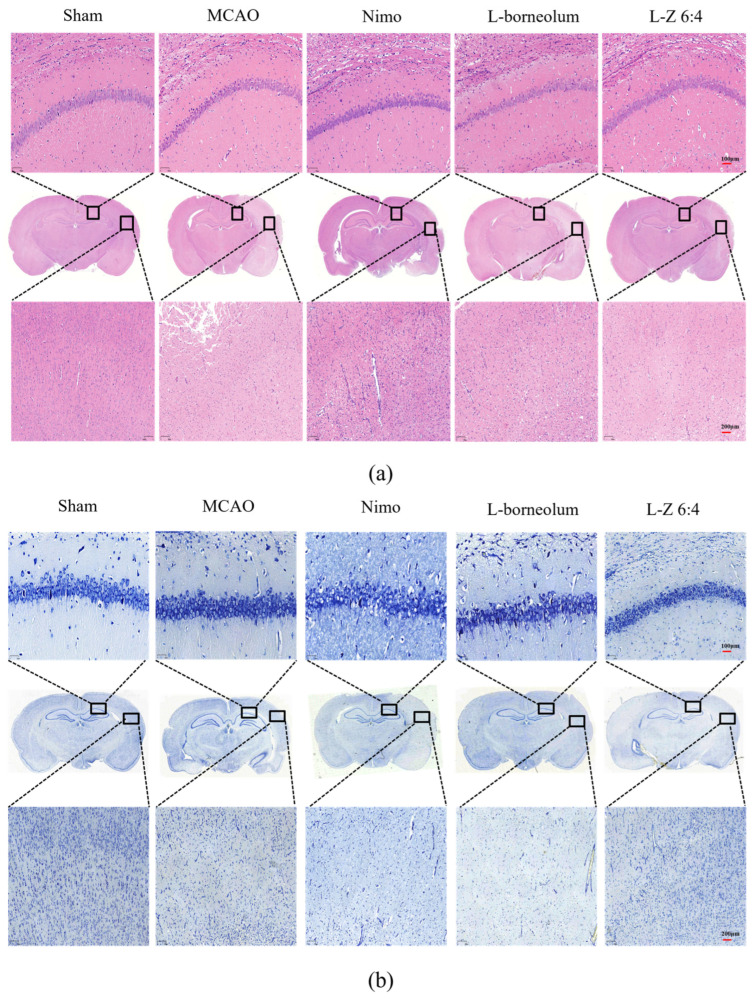
L-borneolum and its main components combination of L-borneol and L-camphor (L-Z 6:4) improved pathological changes in the brain tissue of MCAO rats. (**a**) Pathological changes of hippocampus CA1 (Scale bar, 100 μm) and cortex (Scale bar, 200 μm) by using HE staining. (**b**) Pathological changes of hippocampus CA1 (Scale bar, 100 μm) and cortex (Scale bar, 200 μm) by using Nissl staining.

HE staining and Nissl staining were applied to observe the pathological changes of the rat brain. As exhibited in Figure 4a, the morphology and structure in the cortex and CA1 area of the hippocampus in the Sham group were basically normal, with no obvious ischemia-hypoxia manifestation. In contrast, the MCAO group exhibited severe neuronal damage, including cell swelling, nuclear fragmentation, inflammatory infiltration, and tissue edema. The other treatment groups displayed reduced pathological changes, especially for the L-Z 6:4 group.

In the Sham group, Nissl staining showed intact bluish-purple neurons with abundant Nissl bodies, while the MCAO group exhibited neuronal atrophy, reduced Nissl bodies, and nuclear deformation (Figure 4b). After oral treatment with Nimo, L-borneolum, L-Z 6:4, both the increased Nissl body density and neuronal integrity in the hippocampal CA1 and cortex were observed. Notably, compared to L-borneolum, L-Z 6:4 groups showed a superior neuroprotective effect, with clearer nuclear boundaries and enhanced neuronal preservation in ischemic cortical regions.

### 3.6. Effect of L-Z on Rats’ BBB Function

The BBB is the first barrier to the homeostasis of the brain. It exerts a pivotal regulatory role in cerebral injury secondary to IS, serving as a critical barrier that prevents pathogenic substances, toxins, and harmful macromolecules in the circulation from infiltrating and inducing damage to brain parenchymal tissue [22,23,24]. It exhibits a close association with the onset, progression, and prognosis of IS [25]. The main cause of BBB leakage caused by the latest discovery is damage to the transcellular barrier [26,27,28]. Related studies have found that the decrease of brain edema, nerve injury score and infarct size in stroke model rats was related to the transcytosis of the BBB by TCM [12]. Therefore, in this study, we further explore the regulating effect of L-borneolum and L-Z on the BBB to reveal the mechanism of the neuroprotective effect after IS.

**Figure 5:**
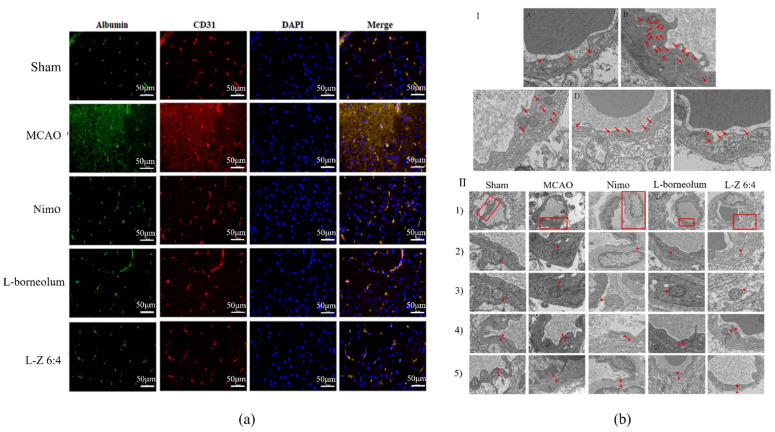
L-borneolum and its main components combination of L-borneol and L-camphor (L-Z 6:4) improved the BBB transfer function. (**a**) Albumin leakage fluorescence staining image (Scale bar, 50 μm). (**b**) Ⅰ, Transport function of the capsule cellar on the BBB. Ⅱ, the BBB ultrastructure after 6 h of MCAO reperfusion. (1). Cross-section of BBB, pericytes in red box; (2). pericytes; (3). mitochondria within pericytes; (4). Tight junctions (TJs); (5). Basement membrane. Scale bar, 500 nm.

As shown in Figure 5a, albumin and CD31 protein expression was significantly increased in the MCAO group compared with the sham group, suggesting marked albumin leakage on the ischemic side. In contrast to the MCAO group, both protein levels were reduced in treatment groups, and the L-Z group exhibited the minimal expression of these two proteins. TEM analysis revealed that the number of vesicles was increased and the structure of pericytes and BEM tight junctions (TJs) in the cortex was damaged in MCAO group, whereas L-borneolum and L-Z treatment attenuated the TJs and other damage (Figure 5b). These results suggest that L-borneolum and L-Z intervention can improve brain injury after acute IS by inhibiting the transcellular transport of the BBB.

### 3.7. Effects of L-Z on hCMEC/D3 Cell Viability

**Figure 6:**
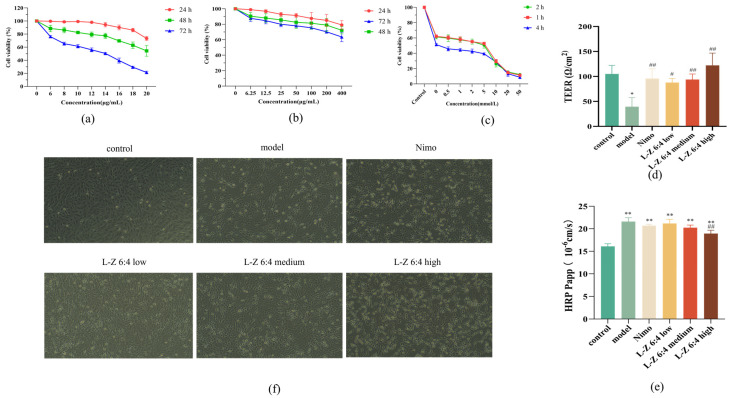
Effect of L-Z on hCMEC/D3 cell viability and BBB permeability. (**a**) Effect of L-Z 6:4 on hCMEC/D3 cell viability. (**b**) Effect of nimodipine on hCMEC/D3 cell viability. (**c**) Effect of Na2S2O4 on hCMEC/D3 cell viability. (**d**) TEER value of cell co-culture model (**e**) Effect of L-Z 6:4 on the morphology of hCMEC/D3 cell. (**f**) Papp value of HRP on the cell co-culture model. * *p* < 0.05, ** *p* < 0.01 vs. control, # *p* < 0.05, ## *p* < 0.01 vs. model.

L-Z had no obvious toxic effects on hCMEC/D3 cells (Figure 6c). According to the experimental requirements, the time of administration was determined to be 24 h. L-Z was administered at three concentration gradients: 50 μg/mL (low), 100 μg/mL (medium), and 200 μg/mL (high). Nimodipine at a concentration of 20 μg/mL was employed as the positive control drug (Figure 6b). Na_2_S_2_O_4_ was able to significantly damage hCMEC/D3 cells compared with the control group and DMEM high glucose medium (Figure 6c). It was concluded that 5 mmol/L concentration for 2 h was used as the final OGD condition.

### 3.8. Effects of L-Z on BBB Permeability of hCMEC/D3 Cell

After OGD injury, the TEER of the model group was markedly reduced compared with the control group (*p* = 0.0023), In contrast, no statistically significant fluctuations in TEER were observed in the Nimo group and the L-Z 6:4 groups treated with different doses (*p* = 0.9456; *p* = 0.6332, *p* = 0.8895, *p* = 0.6332). Relative to the model group, all dosing groups exhibited a significant elevation in TEER (*p* = 0.0069; *p* = 0.0191; *p* = 0.0089; *p* = 0.0003) (Figure 6d). Collectively, these findings confirmed the successful establishment of the in vitro blood-brain barrier (BBB) model in the present study.

Microscopic observation showed that the cells in the control group grew densely, with a spindle shaped morphology and tightly arranged cells. In the model group, some of the cells were seen to be wrinkled and there were gaps between the cells with poor morphology. The cell morphology of the model group was changed, which indicated that OGD had caused damage to hCMEC/D3 cells. Whereas the Nimo group and three concentrations of L-Z groups exhibited an improved effect on the cell morphology (Figure 6e).

As shown in Figure 6f, after OGD injury, the Papp values of HRP in the model group were significantly higher compared with the control group (*p* < 0.00001). It indicated that the integrity of the BBB model after OGD injury was broken, and the permeability was increased. Compared with the model group, the Papp values of HRP in the Nimo group, the L-Z 6:4 low and medium concentration group tended to decrease but did not significantly change (*p* = 0.6001, *p* = 0.9746, *p* = 0.2290). However, the Papp of HRP in the high concentration group was significantly decreased (*p* = 0.0048). It suggested that the HRP transmittance rate was decreased after the pretreatment with L-Z, and the BBB permeability was decreased.

### 3.9. Effect of L-Z on the mRNA and Protein Expression of Cav-1, MFSD2A, and SREBP1 In Vivo

**Figure 7:**
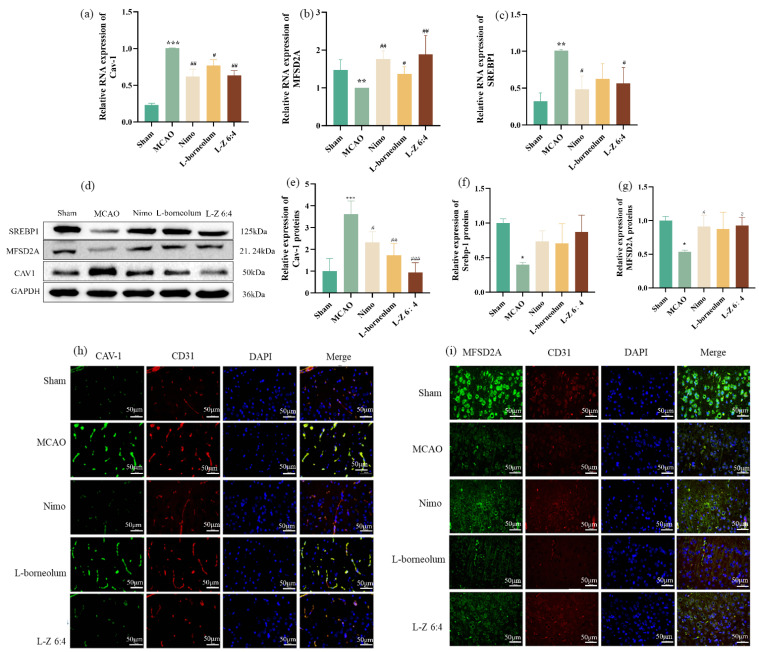
The mRNA and protein expression of CAV-1, MFSD2A and SREBP1 in the brain of rats. $(\bar{x}$ ± S.D, *n* = 3). (**a**–**c**) The mRNA expression of CAV-1, MFSD2A and SREBP-1 by RT-qPCR analysis. (**d**–**g**) The protein expression of CAV-1, MFSD2A and SREBP-1 by Western blot analysis. (**h**) Fluorescence expression of CAV-1 on brain microvascular endothelial cells (Scale bar, 50 μm). (**i**) Fluorescence expression of MFSD2A on brain microvascular endothelial cells (Scale bar, 50 μm). * *p* < 0.05, ** *p* < 0.01, *** *p* < 0.001 vs. Sham group, # *p* < 0.05, ## *p* < 0.01, ### *p* < 0.001 vs. MCAO group.

RT-qPCR assays revealed that the mRNA expression of Cav-1 in the MCAO group was extremely significantly higher (*p* < 0.0001), whereas SREBP1 and MFSD2A mRNA expression levels were markedly reduced (*p* = 0.0030; *p* = 0.3313) compared with the Sham group. The mRNA expressions of Cav-1 in the Nimo, L-borneolum and L-Z 6:4 groups were extremely significantly lower (*p* = 0.0002; *p* = 0.0109; *p* = 0.0003), and the mRNA expression of SREBP1 and MFSD2A in the Nimo, L-borneolum and L-Z 6:4 groups was significantly higher (*p* = 0.389; *p* = 0.2213; *p* = 0.4115/*p* = 0.0524; *p* = 0.5320; *p* = 0.0232) compared with the MCAO group (Figure 7a–c), but no significant difference.

Western blot results indicated that SREBP1 and MFSD2A protein levels were markedly decreased (*p* = 0.0163/*p* = 0.0104), while Cav-1 protein expression was significantly increased (*p* = 0.0005) in the MCAO group relative to the Sham group. Compared with the MCAO group, the protein expressions of MFSD2A were significantly elevated in the Nimo and L-Z 6:4 groups (*p* = 0.0335; *p* = 0.0277). whereas SREBP1 expression showed no significant differences across the Nimo, L-borneol, and L-Z 6:4 groups (*p* = 0.2341; *p* = 0.3145; *p* = 0.0623). Meanwhile, Cav-1 protein expression was significantly reduced in the groups of Nimo, L-borneolum, and L-Z 6:4 (*p* = 0.0459; *p* = 0.0052; *p* = 0.0004) (Figure 7d–g).

Immunofluorescence results showed that the expression of MFSD2A decreased and the expression of Cav-1 increased in the MCAO group relative to the Sham group. The expression of MFSD2A in Nimo, L-borneolum and L-Z 6:4 groups increased, the expression of Cav-1 in Nimo, L-borneolum and L-Z 6:4 groups decreased. Furthermore, the L-Z 6:4 group showing the most significant decrease in Cav-1 expression compared with the MCAO group (Figure 7h,i). It is suggested that Nimo, L-borneolum and L-Z can inhibit MFSD2A/CAV-1 signaling axis on BBB endothelial cells, so that BBB transport function was remodeled, brain injury was slowed down and IS was improved after MCAO.

### 3.10. Effect of L-Z on the mRNA and Protein Expression of Cav-1, MFSD2A, and SREBP1 In Vitro

**Figure 8:**
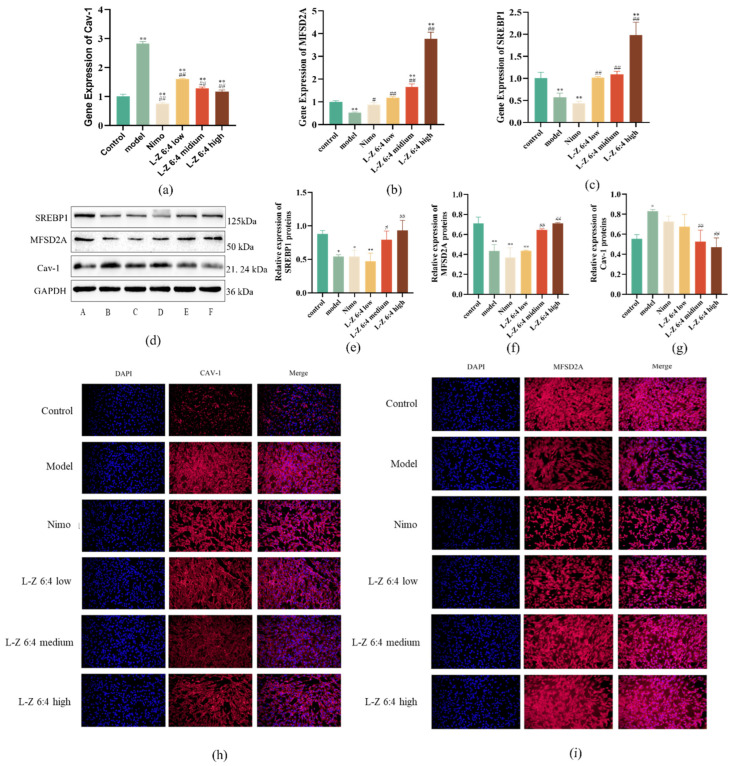
The mRNA and protein expression of CAV-1, MFSD2A and SREBP1 on hCMEC/D3 cells $(\bar{x}$ ± S.D, *n* = 3). (**a**–**c**) The mRNA expression of CAV-1, MFSD2A and SREBP-1 by RT-qPCR analysis. (**d**–**g**) The protein expression of CAV-1, MFSD2A and SREBP-1 by Western blot analysis. A. Control group; B. Model group; C. Nimodipine positive control group; D. L-Z 6: 4 Low-dose group; E. L-Z 6: 4 Medium-dose group; F. L-Z 6: 4 High-dose group. (**h**) Fuorescence expression of CAV-1 on hCMEC/D3 cells. (**i**) Fluorescence expression of MFSD2A on hCMEC/D3 cells. * *p* < 0.05, ** *p* < 0.01 vs. control, # *p* < 0.05, ## *p* < 0.01 vs. model.

According to the RT-qPCR results, compared to the control group, the model group exhibited a significant reduction in MFSD2A and SREBP1 mRNA expression levels (*p* = 0.0018, *p* = 0.0189), whereas CAV-1 mRNA expression was markedly elevated (*p* < 0.0001). In the Nimo group, the mRNA expression levels of CAV-1 and SREBP1 significantly reduced (*p* < 0.0001, *p* = 0.0010), and the mRNA expression of MFSD2A showed no significant change (*p* = 0.8221). By contrast, the high-concentration L-Z 6:4 group displayed a significant upregulation in MFSD2A, CAV-1, and SREBP1 mRNA expression (*p* < 0.0001). Relative to the model group, the Nimo group exhibited a notable restoration of MFSD2A and CAV-1 mRNA expression toward the control group levels (*p* = 0.0239, *p* < 0.0001), whereas SREBP1 mRNA expression showed a downward trend without reaching statistical significance (*p* = 0.8548). In all L-Z 6:4 concentration groups, the mRNA expressions of MFSD2A were significantly increased (*p* < 0.0001), the mRNA expressions of SREBP1 increased (*p* = 0.0239, *p* = 0.0084, *p* < 0.0001), while the CAV-1 mRNA expression level was significantly decreased (*p* < 0.0001) (Figure 8a–c).

Western blot analysis revealed that the model group exhibited significantly decreased protein expression of MFSD2A and SREBP1 (*p* = 0.0005, *p* = 0.0188) but increased protein expression of CAV-1 (*p* = 0.0156) relative to the Sham group. Both the Nimo and L-Z 6:4 low groups further reduced the protein expression of MFSD2A and SREBP1 (*p* < 0.05). In contrast, no significant impact was detected on CAV-1 protein expression (*p* > 0.05). The L-Z 6:4 medium and high groups significantly upregulated the protein expression of MFSD2A (*p* = 0.0051, *p* = 0.0005) and downregulated the protein expression of CAV-1 (*p* = 0.0082, *p* = 0.0021), while the L-Z 6:4 medium concentration group increased SREBP1 (*p* = 0.1032), and the L-Z 6:4 high group induced a more pronounced the protein expression of SREBP1 upregulation (*p* = 0.0071) compared with the model group (Figure 8d–g).

The results of cellular immunofluorescence showed that the red fluorescence belonging to CAV-1 in the model group and other treatment groups were significantly increased, while the blue fluorescence change of cell nucleus DAPI staining was not significant and the red fluorescence of MFSD2A was significantly weakened compared with the control group. Compared with the model group, the red fluorescence of CAV-1 in the Nimo group and the L-Z 6:4 treatment groups were significantly reduced and showed a concentration dependent relationship. The fluorescence of MFSD2A gradually increased, and the L-Z 6:4 high group had approached the fluorescence intensity of the control group, which was much higher than that of the model group. This suggests that L-Z 6:4 enhances MFSD2A protein expression and reduces CAV-1 protein expression to protect BBB function (Figure 8h,i). This suggests that L-Z 6:4 can protect the BBB by enhancing the protein expression of MFSD2A and reducing the protein expression of Cav-1.

## 4. Discussion

IS disrupts the integrity of the BBB, which increases the risk of brain damage, including neurological deficits, and can even be fatal [8,9,29]. After IS, BBB destruction exacerbates complications such as hemorrhagic transformation, brain edema, neurological dysfunction, and secondary injury [8,9,30,31]. Due to the absence of fenestrations, the vesicle transport rate in cerebrovascular endothelial cells is significantly lower than that of peripheral vascular endothelial cells [32]. Additionally, there are complex and continuous TJs between cerebrovascular endothelial cells that contribute to the unique restrictive properties of the BBB [10]. The low permeability of the BBB primarily depends on TJs pathway and the transcytosis pathway [12,33].

In the past, it was believed that BBB leakage was mainly due to alterations in TJs. Nevertheless, recent studies have demonstrated that the initial indicator of BBB leakage is enhanced vesicle formation between cerebrovascular endothelial cells, while the TJs remain intact [13,14,34]. This implies that even when intercellular vesicle formation increases, TJs can still effectively regulate extracellular substances that cannot be transported via transcytosis. Therefore, the abnormal increase of vesicle transport in BBB is one of the manifestations of BBB dysfunction. In recent years, many scholars have proposed that the inhibition of transcytosis plays a central role in maintaining functional barriers. Consequently, protecting the BBB is increasingly recognized as a crucial strategy for stroke treatment [29]. Recent studies have demonstrated that L-borneolum exhibits anti-ischemic and anti-hypoxic effects on the brain and helps improve BBB integrity [1,2]. L-borneol and L-camphor are the main components of L-borneolum. However, there is limited research on the therapeutic effects of different combinations of these two components in disease contexts. Our previous study found that L-borneol and L-camphor could improve cerebral ischemic injury in permanent middle cerebral artery occlusion (pMCAO) rats, with the L:Z (6:4) ratio showing the most significant neuroprotective effect [5]. Therefore, focusing on the traditional Chinese medicine L-borneolum and the main component combination of L-borneol and L-camphor (L:Z 6:4) [5,35]. We systematically explored its molecular mechanisms in ameliorating IS brain injury by regulating BBB transcellular transport in animal experiments.

Through network pharmacology prediction, it was found that the treatment of ischemic stroke with L-borneol and L-camphor was related to vesicle transport and neurological function protection. It is mainly involved in cellular components such as the axon (GO: 0030424), postsynapse (GO: 0098794) and exocytic vesicle membrane (GO: 0099501). It provides an important reference for our later research. Additionally, targets predicted through network pharmacology (such as SLC6A4, ACE, etc.) may hold relevance to biological studies on the BBB damage, offering valuable insights for subsequent research and adding depth at the mechanistic level.

The rat MCAO model was successfully established. This was confirmedthrough assessments of motor function scores, cerebral blood flow, TTC staining, HE staining and Nissl staining. The model closely mirrored the symptoms observed in patients, and these symptoms were significantly improved following treatment with L-borneolum and its main components, L-borneol and L-camphor. These results indicate that L-borneolum and its L-Z (6:4) formulation can effectively ameliorate IS injury.

After IS, brain microvascular endothelial cells contribute to BBB dysfunction [36], which is a critical factor leading to brain injury and neurological deficits. TEM studies have shown that increased cerebral edema, higher nerve injury scores, and larger infarct area in MCAO model rats correlate with enhanced transcellular transport in brain microvascular endothelial cells [13]. To further investigate whether the regulation of the BBB by L-borneolum and the L-Z (6:4) combination improves brain injury after IS by modulating transcellular transport, the BBB was examined by TEM 6 h after reperfusion following MCAO. Vesicular transport in brain microvascular endothelial cells was significantly upregulated in the MCAO group. Treatment with nimodipine, L-borneolum, or the L-Z (6:4) combination significantly reduced the number of vesicles in endothelial cells compared to the MCAO group. Recent studies suggest that the primary cause of BBB leakage following IS injury is damage to the transcellular barrier [26,27]. The ultrastructural findings in this study align with these previous reports. TEM analysis of the BBB ultrastructure and albumin leakage assays indicated no significant differences in tight junctions (TJs) among the nimodipine, L-borneolum, and L-Z (6:4) groups. Similarly, the basic morphology of pericytes and brain endothelial microvessels showed no significant differences across these groups. These results suggest that nimodipine, L-borneolum, and the L-Z (6:4) combination may ameliorate brain injury after IS by inhibiting transcellular transport across the BBB.

MFSD2A, a transmembrane protein specifically expressed on the luminal membrane of cerebrovascular endothelial cells, maintains low blood-brain barrier permeability by inhibiting transcytosis vesicle trafficking [16]. Sterol Regulatory Element-Binding Protein 1 (SREBP1) is a pivotal transcription factor that predominantly governs the de novo synthesis of fatty acids and triglycerides. SREBP1 directly initiates MFSD2A transcription by virtue of binding to the sterol regulatory element within the MFSD2A gene promoter region, thereby increasing its mRNA and protein expression levels. Previous studies have demonstrated that SREBP1 regulates MFSD2A expression, and both proteins participate in brain development and BBB function regulation through lipid metabolism pathways, providing a theoretical foundation for this research [37]. When MFSD2A was absent, the count of Cav-1-positive vesicles in cerebrovascular endothelial cells increased, and BBB permeability was significantly elevated, but TJs were not affected, suggesting that BBB disruption was mainly caused by transcytosis abnormalities [12]. CD31 is a vascular endothelial cell digitizing protein that can be used to localize endothelial cells. Fluorescence co-localization showed that the expression of MFSD2A/Cav-1 signaling axis on cerebral microvascular endothelial cells in the model group after MCAO was altered accordingly, and the results showed that the Nimo, L-borneolum, and L-Z (6:4) groups inhibited the MFSD2A/Cav-1 signaling axis on the BBB endothelial cells. The BBB transport function was remodeled, slowed down the cerebral injury after MCAO, and improved the IS.

**Figure 9:**
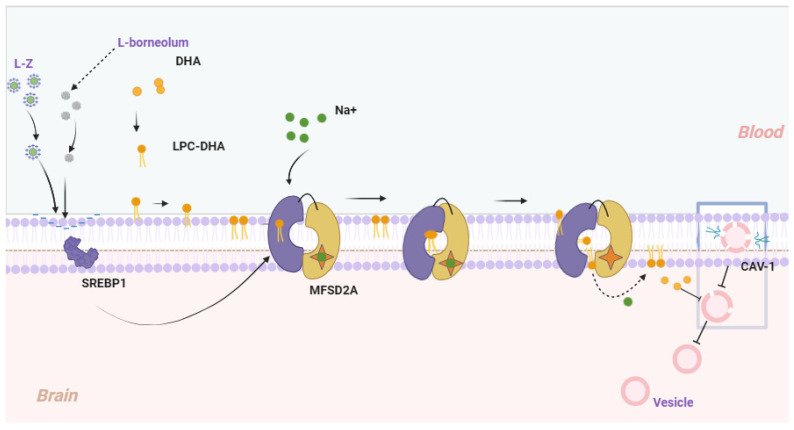
Mechanistic map of the MFSD2A/Cav-1 signaling pathway regulating BBB permeability.

This study is the first to suggest that L-borneolum and its main compounds (L-borneol and L-camphor combination) are associated with the MFSD2A/Cav-1 signaling axis on BBB endothelial cells, thereby improving the BBB transporter function and attenuating the brain injury after MCAO in rats. In this study, the L-Z 6:4 group was found to have a synergistic effect in protecting the rat brain from MCAO surgery injury compared with the drug alone. As a traditional Chinese medicine, the main components of L-borneolum (L-borneol and L-camphor) were more associated with regulating the MFSD2A/Cav-1 signaling axis on the BBB. Therefore, the BBB co-culture model was established in vitro to verify the cerebroprotective effects of the main components of L-borneolum on IS in the MFSD2A/CAV-1 signaling pathway. RT-qPCR analyses demonstrated that the expressions of MFSD2A and SREBP1 mRNA were substantially elevated to different degrees in each administration group, while CAV-1 mRNA expression exhibited a marked decrease in comparison to the model group. Western blot assays demonstrated that a pronounced increase (*p* < 0.05) in the protein levels of MFSD2A and SREBP1, alongside a substantial reduction (*p* < 0.01) in CAV-1 protein expression in both the medium and high concentration groups of L-Z 6:4 when compared to the model group. The results of cellular immunofluorescence showed that the red fluorescence of CAV-1 in the Nimo group and each L-Z 6:4 dosing group was significantly weakened, the fluorescence of MFSD2A was gradually enhanced, and the fluorescence intensity of the high concentration of L-Z 6:4 was already close to that of the control group, which was much higher than that of the model group. These results indicated that SREBP1 could increase the expression of MFSD2A, and MFSD2A inhibited the expression of CAV-1, which might be the mechanism of action of the cerebroprotective effect of L-Z 6:4 (Figure 9). This result is consistent with previous network pharmacology predictions and in vivo animal experiments. However, the current research on the MFSD2A/Cav-1 signaling axis is limited to its association with a variety of ischemia and hypoxia-related barrier disorders. The specific role of upstream and downstream proteins is still unclear, which requires further study.

## 5. Insufficient Experiments and Limitations

A key limitation is the constrained sample size in mechanistic assays, which may restrict the generalizability of secondary findings. However, sample size was rigorously determined per the 3R principles and ARRIVE guidelines, via pilot experiments and statistical power analysis, to ensure statistical validity while minimizing animal usage.

Notably, our core conclusions on the SREBP1/MFSD2A/Cav-1 signaling axis are robustly supported by consistent results from complementary in vivo, in vitro, and network pharmacology predictions, which collectively mitigate the impact of limited sample size in individual experiments.

In addition, this study has several shortcomings. First, the mechanism underlying the neuroprotective effect of L-Z 6:4 remains incompletely elucidated. While this study has established that the neuroprotective action of L-Z 6:4 is implicated in the activation of the MFSD2A signaling pathway, exploration of upstream and downstream proteins is insufficient, with only SREBP1 and CAV1 identified. Further investigation is required. Second, this study did not employ counter-experimental methods such as gene silencing or knockout to validate proteins associated with the MFSD2A pathway, necessitating further experimental validation. Third, the in vitro experiments were conducted solely using a tri-cell co-culture model, which cannot measure in vivo DHA levels. In vivo assays are needed to detect brain DHA levels and verify the expression of proteins related to the MFSD2A signaling pathway. Integrating in vivo and in vitro approaches would provide stronger evidence for the mechanism underlying L-Z 6:4 neuroprotective effects.

## 6. Conclusions

In summary, L-borneolum and its main components (a combination of L-borneol and L-camphor, 6:4 ratio) both effectively improved neurological function and cerebral infarction area. Its mechanism may be related to regulating the BBB’s structure and function by affecting the MFSD2A/CAV-1 pathway. This investigation provided a novel insight into the use of the main components of L-borneolum for the treatment of IS and highlights the importance of BBB function in this disease.

## Figures and Tables

**Table 1:** The standard for the scores of Zea Longa and mNSS.

	Standard	Scores
Zea Longa	Normal rats	0
	Front paws cannot be extended	1
	Rats collapse to the uninjured side	2
	Rats rotate in circles towards the uninjured side	3
	Rats without autonomous walking awareness	4
mNSS	Normal rats	0
	Mild injury	1–6
	Moderate impairment	7–12
	Severe impairment	13–18

**Table 2:** Primer sequences.

Gene Symbol	Sequence	Bps
SREBP-1	F: CGC TAC CGT TCC TCT ATC AAT GAC	24
	R: AGT TTC TGG TTG CTG TGC TGT AAG	24
CAV-1	F: AAG ATG TGA TTG CGG AAC CAG AAG	24
	R: ATA GGG ATG CCG AAG ATG GTA GAC	24
MFSD2A	F: ATG GCT CCT ATC ATC CTC ATC CTG	24
	R: CTT GTT CTG TCG CCG CTT CTC	21
GAPDH	F: GAC ATG CCG CCT GGA GAA AC	20
	R: AGC CCA GGA TGC CCT TTA GT	20

## Data Availability

Due to ethical restrictions imposed by the Institutional Animal Care and Use Committee (IACUC) of Shaanxi University of Chinese Medicine and compliance with the ARRIVE guidelines for animal research, the raw data generated in this study (including animal survival data, neurobehavioral test results, histological images, and molecular biology assay data) contain sensitive information related to experimental animal welfare and protocol-specific details. These data are not publicly available to prevent potential misuse or misinterpretation that could compromise the integrity of animal research ethics. Upon reasonable request, de-identified data (with all animal-specific identifiers removed) may be made available to qualified researchers for non-commercial, scientific research purposes. Requests should be directed to the corresponding author (Taiwei Dong, dongtaiwei2023@163.com) for review and approval. All data sharing will adhere to institutional ethical policies and applicable regulations governing animal research data management.

## Abbreviations

The following abbreviations are used in this manuscript:

IS	ischemic stroke
MCAO/R	middle cerebral artery occlusion/reperfusion
TTC	2,3,5-Triphenyl tetrazolium chloride
HE	Hematoxylin-eosin
TEM	transmission electron microscopy
BBB	blood-brain barrier
MTT	3-(4,5-dimethylthiazol-2-yl)-2,5-diphenyltetrazolium bromide
TEER	Trans-epithelial electrical resistance
HRP	Horseradish peroxidase
RT-qPCR	Real-time fluorescent quantitative PCR
LPC	Lysophosphatidylcholine transporter
DHA	Docosahexaenoic Acid
CNS	central nervous system
TJs	tight junctions
SDF	Standard Database Format
PPI	protein-protein interaction
CCA	common carotid artery
ECA	external carotid artery
ICA	internal carotid artery
PFA	4% paraformaldehyde
OD	optical density
BSA	bovine serum albumin
MFSD2A	Major Facilitator Superfamily Domain Containing 2A
CAV-1)	caveolin-1
SREBP1	Sterol Regulatory Element-Binding Protein 1
GAPDH	glyceraldehyde-3-phosphate dehydrogenase
OCT	optimal cutting temperature
DAPI	4′,6-Diamidino-2′-phenylindole
TVS	tab-separated values
Papp	Apparent permeability coefficient
mNss	modified neurological severity score
